# Development and Evaluation of Drug Loaded Regenerated Bacterial Cellulose-Based Matrices as a Potential Dosage Form

**DOI:** 10.3389/fbioe.2020.579404

**Published:** 2020-12-03

**Authors:** Munair Badshah, Hanif Ullah, Feng He, Fazli Wahid, Umar Farooq, Mattias Andersson, Taous Khan

**Affiliations:** ^1^Department of Pharmacy, COMSATS University Islamabad, Abbottabad, Pakistan; ^2^Hubei Key Laboratory of Economic Forest Germplasm Improvement and Resources Comprehensive Utilization, Huanggang Normal University, Huanggang, China; ^3^Department of Biomedical Sciences, Pak-Austria Fachhochschule: Institute of Applied Sciences and Technology, Haripur, Pakistan; ^4^Department of Chemistry and Chemical Engineering, Chalmers University of Technology, Gothenburg, Sweden

**Keywords:** bacterial cellulose, drug delivery, famotidine, tizanidine, regeneration

## Abstract

Bacterial cellulose (BC) is a highly pure form of cellulose and possesses superior physico-mechanical properties with wide range of applications. These properties of BC can further be improved by various modifications, including its regeneration from the BC solution. In the current research work, regenerated BC (R-BC) matrices were prepared using N-methyl-morpholine-oxide (NMMO; 50% w/w solution in water) and loaded with model drugs, i.e., famotidine or tizanidine. The characterization of drug loaded regenerated BC (R-BC-drug) matrices was carried out using Fourier transform infrared spectroscopy (FTIR), x-ray diffraction (XRD) analysis, scanning electron microscopy (SEM) and thermogravimetric analysis (TGA), which revealed the stability of matrices and successful drug loading. Results of dissolution studies showed immediate (i.e., >90%) drug release in 30 min. The drugs release data was found to best fit into first order kinetics model having *R*^2^ values >0.99 for all the formulations. These results indicated that regenerated BC-based matrices had the ability to be used for delivery of orally administered drugs.

## Introduction

Cellulose is the most abundant, inexpensive, biodegradable, and renewable biomaterial obtained from cotton, wood, and other plant sources, which often contains pectin, lignin, and hemicellulose as biogenic contaminations (Khan et al., 2015; Hickey and Pelling, 2019). In contrast, bacterial cellulose (BC) is highly pure, non-cytotoxic and non-pyrogenic biomaterial, obtained by culturing the colonies of selected bacterial species, particularly *Gluconacetobacter xylinus* (*G. xylinus*) (Ávila et al., 2015; Ullah et al., 2016a). The features like molding ability into the desired shape, ultrafine, and well-organized fibrous network, and ability of absorption and retention of water make BC superior to plant based cellulose (Sajjad et al., 2020; Souza et al., 2020). BC finds applications in the preparation of medical devices, tissue engineering and reconstructive surgery, and as supportive scaffold for reconstruction of auricular cartilage (Ávila et al., 2015; Möller et al., 2017). In addition, BC has been studied for potential applications, such as tissue proliferation, cell growth, treatment of wounds, mesenchymal cells differentiation, enzymes and bioactive compounds delivery (Klinthoopthamrong et al., 2020), and as-synthesized as well as surface modified matrices (Badshah et al., 2017,18) and capsules shells for oral drug delivery (Ullah et al., 2017).

To meet the current research demand and to explore further potential applications of BC in various fields, several physical and chemical procedures have been reported in the literature for preparation of BC based nano-composites (Ullah et al., 2016a,b) and bio-functionalized polymers (Badshah et al., 2018). Out of these methods, the most popular are the surface modification, and novel dissolution and regeneration process (Khalid et al., 2017; Ullah et al., 2019). Limited data is available regarding biomedical and pharmaceutical applications of regenerated bacterial cellulose (R-BC). The applications of R-BC include microfluidic column for protein separation, electricity conducting multi-walled carbon nano-tubes (Phisalaphong et al., 2008; Chen et al., 2016), novel nano-porous membrane for filtration and diode for light emission (Chen et al., 2009, 2016). In addition, R-BC has been studied as wound dressing material for the delivery of nanoparticles, as scaffold for tissue regeneration (Ul-Islam et al., 2014), delivery of antibacterial agents and for biomedical tissue engineering (Shafiei et al., 2014; Reddy and Yang, 2015). In addition to above-mentioned applications, BC forms a single excipient based intact oral dosage form due its higher tensile strength in comparison to the existing conventional solid dosage forms. Moreover, the as-synthesized BC membrane has limited thickness and more time is required to obtain desired thickness. In case of regenerated BC, the matrices with desired thickness can be easily produced by increasing the quantity or concentration of BC solution (Badshah et al., 2017, p. 18; Ullah et al., 2017). Similarly, R-BC is more amorphous and easily biodegrade in comparison to as-synthesized BC and thus have high potential for drug delivery to the body parts, wherein degradation of BC is desired (Ullah et al., 2019).

The process of BC regeneration is associated with certain shortcomings, such as its limited solubility in solvents (commonly used for plant cellulose) and inability to tailor the polymeric properties after regeneration (Reddy and Yang, 2015). The literature studies showed that N-methyl-morpholine-oxide (NMMO) is an environment friendly and recyclable solvent (Shafiei et al., 2014; Reddy and Yang, 2015), and possesses better cellulose dissolution properties as compared to other chemicals (El-Wakil and Hassan, 2008; Xu et al., 2015). The use of NMMO revolutionized the regeneration process of cellulose, and expanded its applications from textiles and cotton fiber spinning industry (Xu et al., 2015) to biomedical field (Isogai and Atalla, 1998; Zhu et al., 2006). Literature revealed the availability of several studies on the dissolution and regeneration of plant based cellulose, but little data is available related to BC regeneration. In addition, no studies have been reported regarding R-BC applications as matrices for oral delivery of drugs. Therefore, the current research was designed to dissolve and regenerate BC for drug delivery using NMMO as solvent.

In the current research work, R-BC-drug (famotidine and tizanidine) matrices were prepared for the first time using NMMO as solvent. The matrices were characterized using FTIR, XRD, SEM and TGA. The matrices were evaluated (*in-vitro)* using simulated gastric conditions with objective to explore the possible applications of R-BC in oral drug delivery.

## Materials and Methods

### Materials

Anhydrous D-glucose (Dae-Jung, Gyeonggi-do, Korea), agar and peptone (Oxide, Hants, UK), sodiumhydroxide (Sigma Aldrich, St. Louis, USA), citric acid monohydrate (RDH, Seelze, Germany), sodium dihydrogen phosphate and yeast extract (Merk, Darmstadt, Germany), NMMO 50% (w/w) aqueous solution (a kind gift from Amines and Plasticizer, Mumbai, India), tizanidine HCl (JPN Pharma, Mumbai, India), famotidine (Suleshvari Pharma, Gujarat, India), and hydrochloric acid (Fishers Chemicals Ltd, Loughborough, UK) were used. The solvents and chemicals received were used without further processing.

### Methods

#### Biosynthesis of BC

Hestrin Schramm (HS) liquid medium, containing glucose anhydrous 2% citric acid monohydrate 0.11%, yeast extract 0.5%, peptone 0.5%, NaH_2_PO_4_ 0.27% and distilled-water, was prepared (pH 6.0) and sterilized (121°C for 20 min). For the preparation of the pre-culture, the colonies of *G. xylinus* were added into HS medium (50 mL) in conical flask (100 mL) and incubated for 24 h at 30°C and 150 rpm in shaking orbital incubator (J.P. Selecta S.A, Spain). Then, 20 mL of the pre-cultured medium was added into basal HS medium in a rectangular container (6 cm × 24 cm × 18 cm) and kept in static incubator (Memmert, 100–800, Germany) at 28°C for 7 to 15 days. The prepared BC sheets was thoroughly washed using distilled water followed by addition of 0.3 M sodium hydroxide and sterilized at 121°C for 20 min, to remove culture medium remnants and bacterial colonies. Then, distilled water was used for complete washing of BC till the pH became neutral and stored in distilled water at 4°C for further use (Badshah et al., 2017; Ullah et al., 2017).

#### Preparation of Regenerated Bacterial Cellulose Drug Matrices

**Figure 1:**
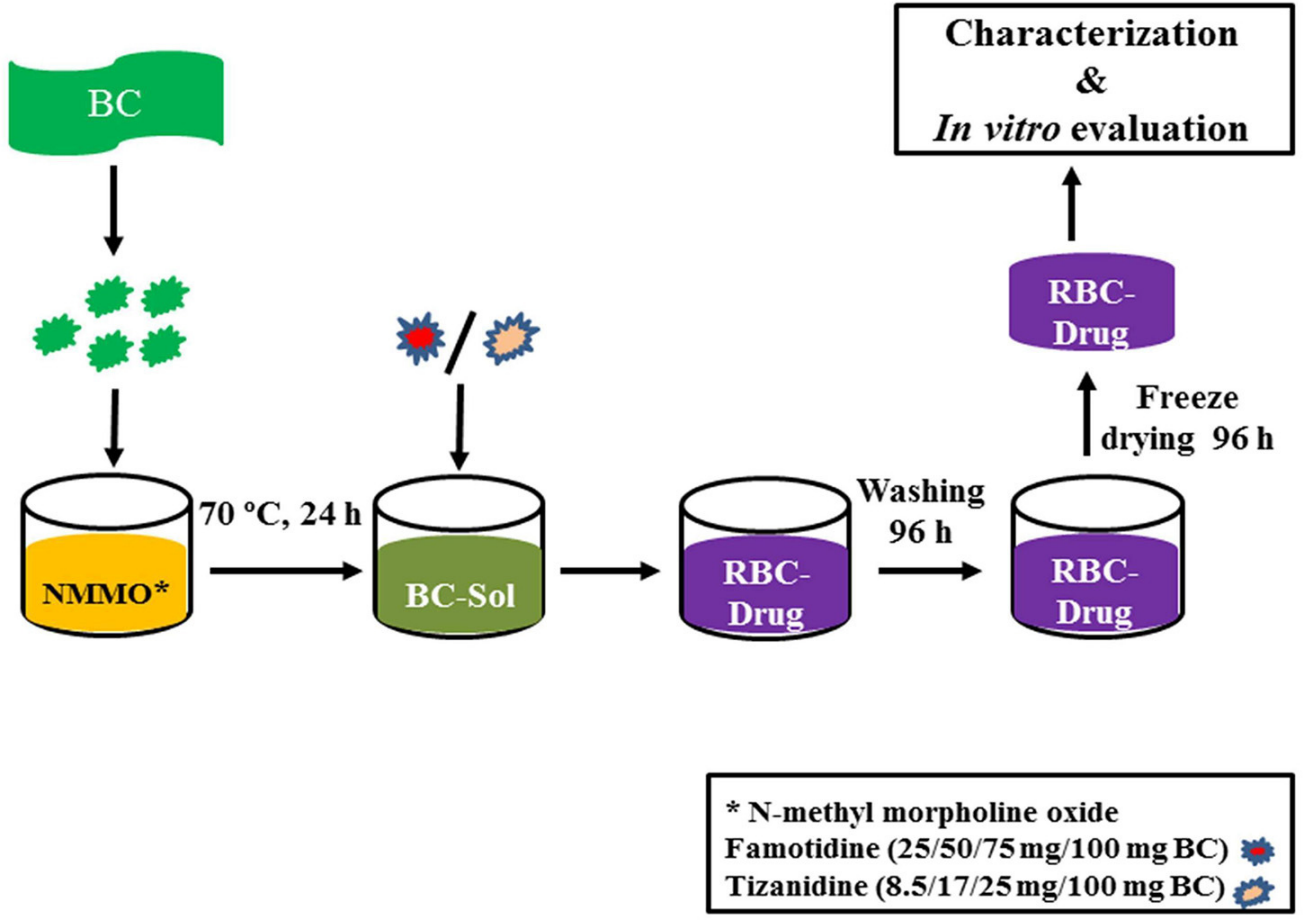
Schematic diagram showing the general process of BC regeneration, drug loading, and matrices preparation.

BC was dried at 60°C for 10 h in a heating oven (SANFA, DHG-9202, Jiangsu Jinyi, China), followed by grinding to convert it into powder form. Then, powdered BC (2 g) was gradually added to NMMO solution (50 g) in glass petri plates (90 mm × 10 mm) and heated at 70°C for 24 h to dissolve it. The BC solution was added with different concentrations of famotidine and tizanidine (Table 1) and thoroughly stirred until dispersed uniformly. The BC-drug mixtures were allowed to stand at room temperature (25°C) until solidification (6 h). In order to remove NMMO, the R-BC-drug sheets were incubated in 50 mL distilled water for 96 h with regular replacement of fresh washing medium each 24 h (4 times) and finally freeze-dried (FreeZone 6, catalog No. 7752030, LABCONCO, USA) at −33°C and 0.025 mbar (Ul-Islam et al., 2014; Khan et al., 2015). Round shaped matrices were prepared from the R-BC-drug sheets using a specially designed fabricator with 12 mm diameter. Figure 1 illustrates the important steps used in the preparation of R-BC-drug matrices.

**Table 1:** Summary of R-BC-drug matrices thickness, friability and drug loading data.

**Formulation**	**R-BC : Drug (g)**	**Thickness (mm) (*n* = 3)**	**Friability (*n* = 20)**	**Drug Loading (%) (*n* = 3)**
F1	1 : 0.25	3.50	0	22.97 ± 0.81
F2	1 : 0.50	3.25	0	24.62 ± 3.98
F3	1 : 0.75	3.20	0	27.70 ± 3.24
G1	1 : 0.085	2.50	0	17.65 ± 1.80
G2	1 : 0.17	2.45	0	24.79 ± 3.27
G3	1 : 0.25	2.65	0	28.32 ± 1.00

### Characterization

#### Fourier-Transform Infrared Spectroscopy

The samples were dried at 50°C for 24 h prior to measurement. FTIR spectra for R-BC, drugs and R-BC-drug matrices was recorded using FTIR spectrophotometer (Perkin-Elmer Frontier FTIR Spectrometer, USA) in the spectral range of 4,000–400 cm^−1^ at resolution rate of 4 cm^−1^, with an ATR Pike Gladi ATR diamond crystal.

#### X-Ray Diffraction Analysis

XRD measurements were employed by means of x-ray diffractometer (D8 ADVANCE, BRUKER, Co. USA) with radiation Cu Kα at 2.29 Å and operated at room temperature for the determination of crystallinity of R-BC, drugs and R-BC-drug matrices. The sample scanning speed was 6°/min and the angle for scanning (2θ) used was in the range of 10–60°.

#### Field Emission Scanning Electron Microscopy

The surface morphology analysis of R-BC and R-BC-drug matrices was carried out with field emission scanning electron microscopy (LEO Ultra 55, LEO Electron Microscopy Ltd, Cambridge, UK). The samples for cross section analysis were prepared under the liquid nitrogen conditions. All the samples were fixed on the SEM holder using adhesive tape prior to proceeding for analysis. The samples were exposed for 1 min for sputter coating with gold in an atmosphere provided with Argon (S150B Sputter Coater, Edwards, England) to determine the surface topography and morphology.

#### Thermogravimetric Analysis

Thermal stability analysis of the R-BC-drug matrices was carried out with the help of thermogravimetric analyzer (TGA/DSC 3^+^, Mettler Toledo, UK). Thermogram of samples was attained in the temperature range of 35 to 800°C with an increment of 10°C/min under the nitrogen atmospheric conditions.

#### Thickness and Drug Loading Efficiency (%)

The R-BC-drug matrices thickness was measured using a Vernier caliper (SparkFun, USA). Percent drug loading of the matrices were calculated by averaging the amount of drug released of all the formulations during dissolution. The following equation was used to calculate matrices percent drug loading.

$$\begin{array}{l} Drug\;laoding\;efficiency\;\left(\%\right)\\ \;=\frac{Loaded\;drug\;\left(based\;on\;the\;released\;amount\right)}{Amount\;of\;drug\;feed\;initially}\;\times 100\; \end{array}$$

### Friability Test

Friability test of the matrices was performed using a Friability Tester (FT-L, Galvano Scientific, Pakistan) having speed of 25 rpm and time limit of 4 min (Badshah et al., 2017). The change in weight of the matrices was calculated using the following equation:

$$\begin{array}{l} x=\frac{\left(W1-W2\right)}{W1}\;\times 100 \end{array}$$

Where W1 represents pre-test weight of matrices, W2 denotes the weight of matrices after test and x show the percent weight loss.

### *In-vitro* Drug Release

The release of drugs from the R-BC-drug matrices was performed in simulated gastric conditions, i.e., 0.1 N HCl solution (900 mL) maintained at 37 ± 0.5°C using USP type-I dissolution apparatus (Dissolutest, Prolabo, France). The paddle rotation speed was in tune of 50 rpm. Samples (5 mL) from the medium were withdrawn at designated time intervals and replaced with an equal amount of fresh medium. The amount of drug released was tested using UV-spectrophotometer (Cary 60 UV-Vis, Agilent Technologies, USA) at 265 nm and 320 nm for famotidine and tizanidine, respectively. All the drug release experiments were performed in triplicate. The data obtained was averaged and presented as cumulative percent release vs. time (Badshah et al., 2017, 2018).

### Drug Release Kinetics

The mechanism for drug release from R-BC-drug matrices was studied by applying selected kinetics models including

$$\begin{array}{l} \text{Zero order}\left({\text{Q}}_{\text{t}}={\text{Q}}_{0}+{\text{K}}_{0}\text{t}\right), \end{array}$$

Where “Qt” is the cumulative amount of drug release at time “t”

“Q_0_” is the initial amount of drug at time “0,” “K_0_” is zero order rate constant and “t” is the time.

$$\begin{array}{l} \text{First order}\left(\text{Log }{\text{Q}}_{\text{t}}=\text{Log }{\text{Q}}_{0}+{\text{K}}_{\text{t}}/2.303\right), \end{array}$$

Where “Q_t_” is the cumulative amount of drug release at time “t”

“Q_0_” is the initial amount of drug at time “0,” “K_t_” is first order rate constant and “t” is the time.

$$\begin{array}{l} \text{Higuchi}\left({\text{Q}}_{\text{t}}={\text{K}}_{\text{H}}{\text{t}}^{1/2}\right) \end{array}$$

Where “Q_t_” is the cumulative amount of drug release at time “t” and “K_H_” is Higuchi rate constant and “t” is the time,

and

$$\begin{array}{l} \text{Korsmeyer}-\text{Peppas}\left({\text{Q}}_{\text{t}}/\text{Q}={\text{K}}_{\text{kp}}{\text{t}}^{\text{n}}\right) \end{array}$$

Whereas “Q_t_” is drug cumulative amount released at time “t,” “Q” is the total amount of drug in the dosage form, “K_kp_” is Korsmeyer-Peppas rate constant, “t” is the time and “n” is diffusion or release exponent (Gouda et al., 2017; Ullah et al., 2017).

### Statistical Analysis

The results obtained from three independent replicate experiments were presented as mean ± SD. The results were analyzed using GraphPad Prism 5.0 software (GraphPad Software Inc. USA). The statistical analysis was performed using one way ANOVA with *post-hoc* Tukey's test, keeping the level of significance with probabilities of ^*^p < 0.05, ^**^p < 0.01, and ^***^p < 0.001.

## Results and Discussion

### Preparation of R-BC-Drug Matrices

In the current study, BC was successfully dissolved in NMMO, incorporated with model drugs in various concentrations and finally regenerated. Several solvents can dissolve BC, however, NMMO was selected in the current study because it is recyclable and environment friendly solvent (Gao et al., 2011; Shafiei et al., 2014). The BC dissolution and regeneration require high temperature and longer duration, which have strong impact on the BC intra- and intermolecular hydrogen bonds breaking, crystallinity, surface morphology, thermal stability and mechanical properties (Gao et al., 2011; Xu et al., 2015). Moreover, the R-BC-drug matrices were subjected to various characterization techniques to study the effects of temperature and chemical changes (if any) during this process (Gao et al., 2011 Ul-Islam et al., 2014).

### Physical Evaluation of R-BC-Drug Matrices

The prepared matrices were subjected to various physical tests. The matrices thickness was observed to be directly proportional to the initial concentration of drug for loading (Table 1). The data of friability test showed no weight loss during the test (Chen et al., 2009; Badshah et al., 2017). Similarly, the amount of drug loaded into R-BC was directly proportional to the initial concentration of the drug added to BC solution before the regeneration (Table 1). In case of tizanidine, the drug loading efficiency was directly proportional to the amount of drug used in the loading process. However, in case of famotidine, the drug loading efficiency was almost constant irrespective of the amount of drug used in the loading process. The possible reason for this constant loading might be that a fixed quantity of BC is available for absorption of drug (Sharma et al., 2016) and the drug concentrations beyond the maximum saturation capacity of the BC cannot be absorbed.

### Characterization of R-BC-Drug Matrices

#### Fourier-Transform Infrared Analysis

**Figure 2:**
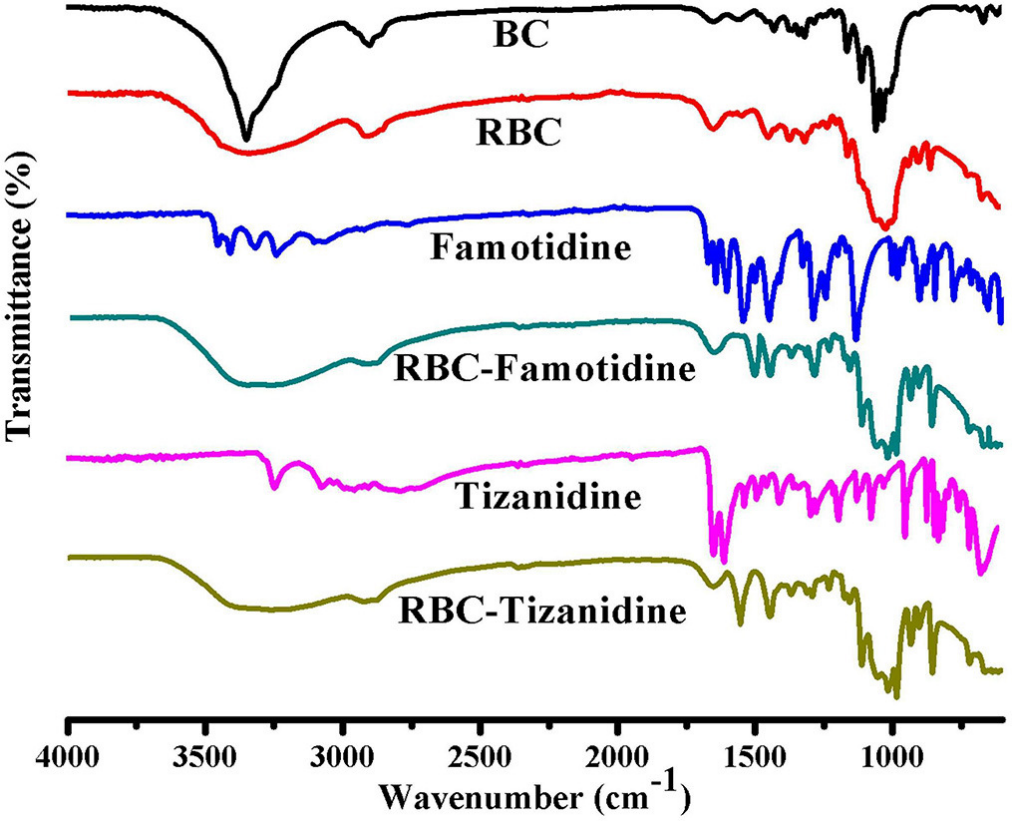
FTIR spectrum of the prepared BC, R-BC, famotidine, R-BC-famotidine, tizanidine, and R-BC-tizanidine matrices for comparison.

FTIR technique was used to study the compatibility and structural changes of the formulations ingredients, i.e., R-BC and drug loaded R-BC. FTIR spectra for BC, R-BC, famotidine, R-BC-famotidine, tizanidine and R-BC-tizanidine have been shown in Figure 2. The spectrum of as-synthesized BC showed characteristic band at 3,500–3,200 cm^−1^ and at 1,160 and 1,068 cm^−1^, which are assigned to OH stretching, OH wagging and C-O-C pyranose ring (Chen et al., 2009; Badshah et al., 2017). The spectrum of R-BC has broad peaks at 3,500–3,200 cm^−1^, representing OH stretching due to breakage of inter and intra molecular hydrogen bonding. In addition, the appearance of peaks in as-synthesized and regenerated BC at 1,160 and 1,068 cm^−1^ represent the C-H scissor vibration (Gao et al., 2011; Shafiei et al., 2014). The IR spectra of R-BC-famotidine revealed a broad band between 3,500 and 3,200 cm^−1^, which may arise due to the merger of –OH and NH_2_ groups of R-BC and famotidine, respectively. Similarly, the region of 2,850–2,950 cm^−1^ represents the C–H bending of R-BC vibration (Sagdinc and Bayari, 2005; Arima and Iwata, 2007). The band at 1,553 cm^−1^ represent NH_2_ group, while at 1,290 and 1,135 cm^−1^ revealed CH_2_=S and SO_2_ groups of famotidine, respectively (Sagdinc and Bayari, 2005; Cheng and Lin, 2008). The peaks at 1,078 and 990 cm^−1^ represent bending vibration due to NH_2_ group of famotidine and CH_2_ group of R-BC (Sagdinc and Bayari, 2005; Souza et al., 2020). The band at 895 cm^−1^ represent glycosidic linkage of R-BC and 850 cm^−1^ has been assigned to the CH_2_ skeleton of famotidine and R-BC (Sagdinc and Bayari, 2005; Gao et al., 2011).

The IR spectrum of R-BC-tizanidine IR displayed bands at 3,200–3,500 cm^−1^ that represent OH and NH_2_ groups of R-BC and tizanidine, respectively. Similarly, bands at 1,665 cm^−1^ indicates the C=C aromatic stretching of tizanidine (Aamir and Ahmad, 2010; Badshah et al., 2018). The peaks at 1,450–1,200 cm^−1^ indicates R-BC C–H stretching vibration. The C–N stretching was confirmed by peak at 1,290 and 1,187 cm^−1^, while bands at 1,113 and 1,068 cm^−1^ confirm the C–Cl group of tizanidine (Aamir and Ahmad, 2010; Badshah et al., 2017, 2018). The presence of characteristic absorption bands of respective drug functional groups with slight shift shows drug incorporation into the R-BC. There has been no new peak appearance in the FTIR spectrum of the respective R-BC-drug composites, which show absence of any chemical boding formation between the components.

#### X-Ray Diffraction Analysis

**Figure 3:**
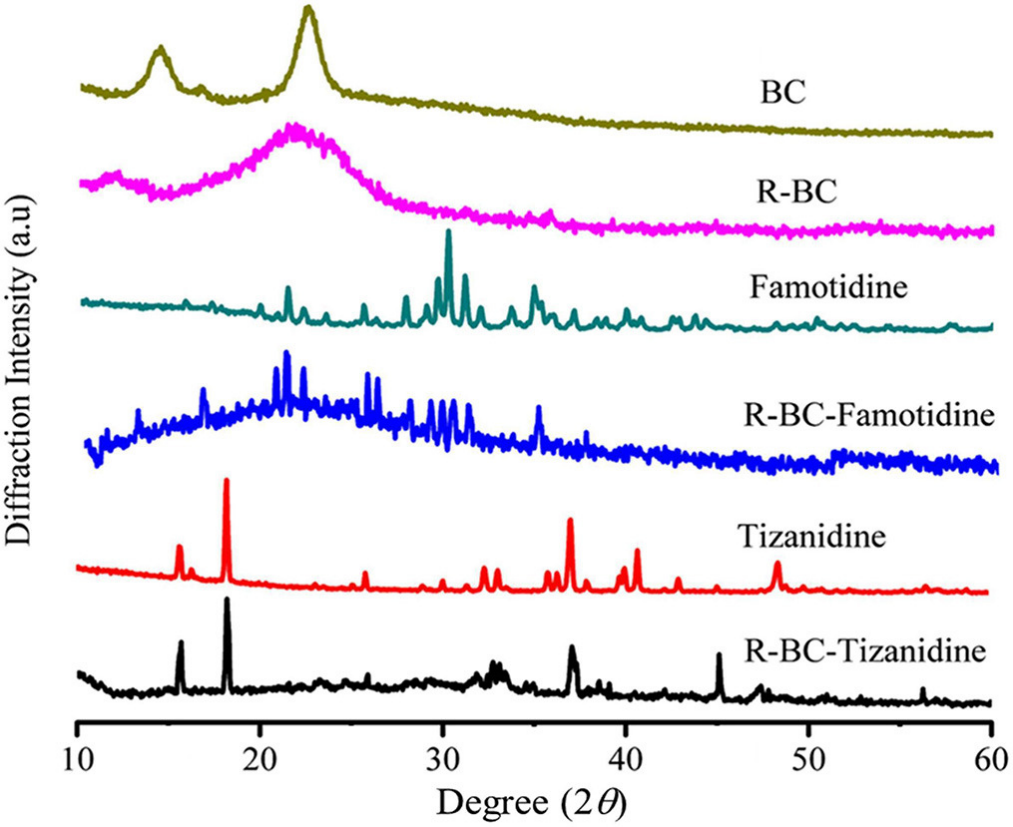
XRD pattern of R-BC, famotidine, R-BC-famotidine, tizanidine, and R-BC-tizanidine.

**Figure 4:**
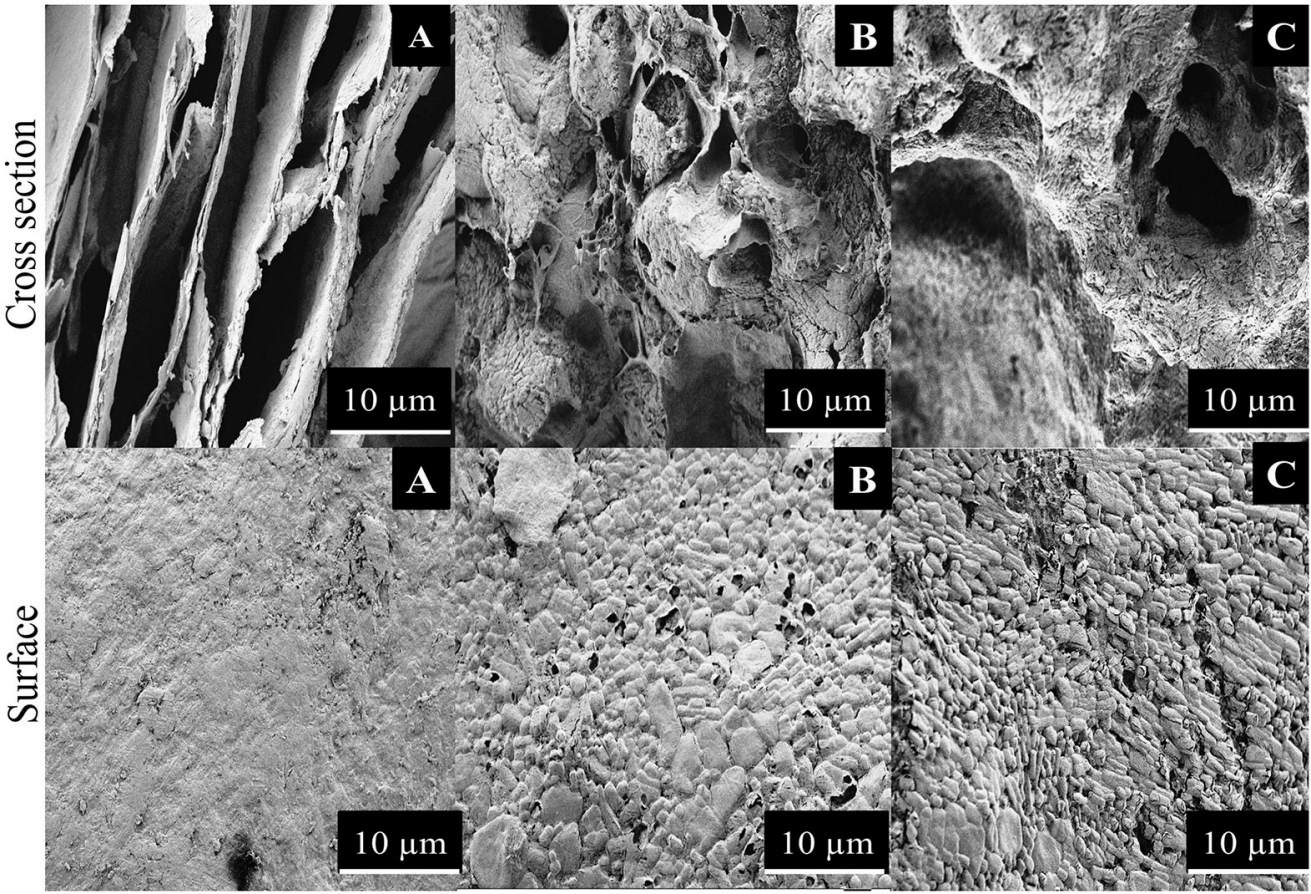
SEM images of **(A)** R-BC, **(B)** R-BC-famotidine, and **(C)** R-BC-tizanidine matrices.

Change in the crystallinity of the R-BC and drug loaded matrices was evaluated using XRD technique. XRD patterns of BC, R-BC, famotidine, R-BC-famotidine, tizanidine HCl and R-BC-tizanidine HCl have been displayed in Figure 3. BC has appearance of characteristic peaks at 14.5, 16.5, and 22.5°. R-BC showed comparative peak broadening at 19.8 and 22.7°, which confirmed the conversion of BC to amorphous form as a result of inter and intra molecular hydrogen bonding disruption during the dissolution process (Lee et al., 2014; Badshah et al., 2017). The pattern for famotidine displayed crystalline nature and the R-BC-famotidine showed few and shorter peaks, which indicated lower crystalline nature and amount of drug loaded. The peaks at 15, 22, 25.6, 30, and 35.5° also showed the presence of famotidine in crystalline form (Razavi et al., 2014). In case of R-BC-tizanidine, distinct peaks at 16, 18.7, and at 32.55, 37.55, and 41.5° with reduced intensity confirmed the presence of tizanidine in crystalline form (Aamir and Ahmad, 2010; Badshah et al., 2017). The presence of the respective drug specified peaks confirmed the effective loadings of drugs into the R-BC-drug matrices in crystalline form. The nanoporous BC can convert the drug into amorphous form when loaded into the pores (Ullah et al., 2019), however, the drug is also present in crystalline form on the surface of matrices due to excessive loading, which is further confirmed by the SEM analysis as shown in Figure 4.

#### Scanning Electron Microscopy

Surface morphology of R-BC and the matrices with loaded drugs was studied using SEM. The SEM micrographs of surface and cross section of R-BC and R-BC-drugs matrices were obtained at varying magnifications. Figure 4 shows the typical microphotographs for R-BC, R-BC-famotidine and R-BC-tizanidine. The cross-section for R-BC (Figure 4A) showed porous layers of different dimensions. These pores may be attributed to the water crystallization during R-BC regeneration process (El-Wakil and Hassan, 2008; Xu et al., 2015). The cross sectional micrograph for the R-BC did not show formation of fibrous structure, which is the confirmation of the native BC fiber network destruction/annihilation during the regeneration process. Similarly, the surface morphology of the R-BC also showed smooth appearance and absence of fibrous network, which confirmed the dissolution of BC followed by regeneration (Wan et al., 2006; Ul-Islam et al., 2014). The cross sectional images of the R-BC famotidine (Figure 4B) and R-BC-tizanidine (Figure 4C) showed comparatively less porous structure than pure R-BC. The possible reason may be that the loaded drugs have filled most of the spaces produced. In addition to this, the probable reason of pores formation may be the wash out of some of the loaded drug during the solvent exchange of NMMO with deionized water and the forced migration of the loaded drug content to the surfaces during the freeze drying of the matrices. The micrographs revealed the presence of drug crystals on the surface of the R-BC film, which confirms successful drug loading.

#### Thermogravimetric Analysis

**Figure 5:**
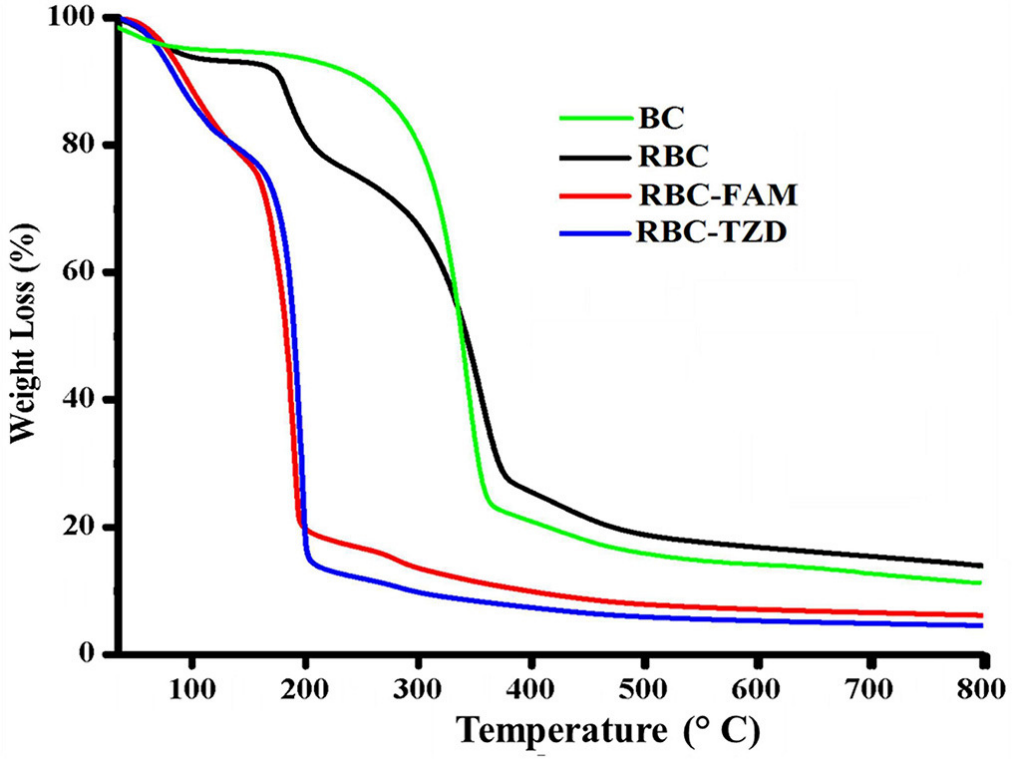
Curves of the thermogravimetric analysis for the prepared matrices of R-BC, R-BC-famotidine, and R-BC-tizanidine.

In order to study the thermal stability of R-BC and drugs loaded matrices, thermal analysis was carried out. Thermogram of BC, R-BC, and R-BC-drugs matrices were obtained in order to study the thermal behavior of these samples (Figure 5). In the initial phase of degradation, BC showed 5% weight loss at temperature up to 280°C possibly due to evaporation of adsorbed water. In the second phase, there is a sharp decline in weight (70%) at a temperature between 280 and 380°C due to breakdown of glycosidic linkages. The rest of the content (25%) remained as ash till 800°C. These results are in agreement with the previous studies (Chen et al., 2016). Similarly, the R-BC displayed 5% weight loss up to 220°C followed by a sudden decline (70%) due to depolymerization and breaking down of glycosidic backbone in the temperature range of 220–380°C. The remaining portion (25%) existed as ash till 800°C. These results were also consistent with the reported thermal studies for regenerated BC (El-Wakil and Hassan, 2008; Gao et al., 2011). On the other hand, R-BC-famotidine and R-BC-tizanidine displayed 6 and 8% loss in weight up to 100°C, respectively. This weight loss may be due to evaporation of surface adsorbed water molecules in R-BC-drug matrices (Badshah et al., 2017). With the rise in temperature from 100 to 200°C, the weight loss count for both drugs of matrices was 80% and the possible reason may be the elimination of hydroxyl groups and combustion of the organic part of R-BC. In addition, the melting temperature of the loaded drug might have caused the abrupt weight loss (El-Wakil and Hassan, 2008; Gao et al., 2011). The data showed that R-BC-famotidine and R-BC tizanidine was maximally degraded (marked as ash) till 800°C (Badshah et al., 2017).

### *In-vitro* Drug Release Studies

**Figure 6:**
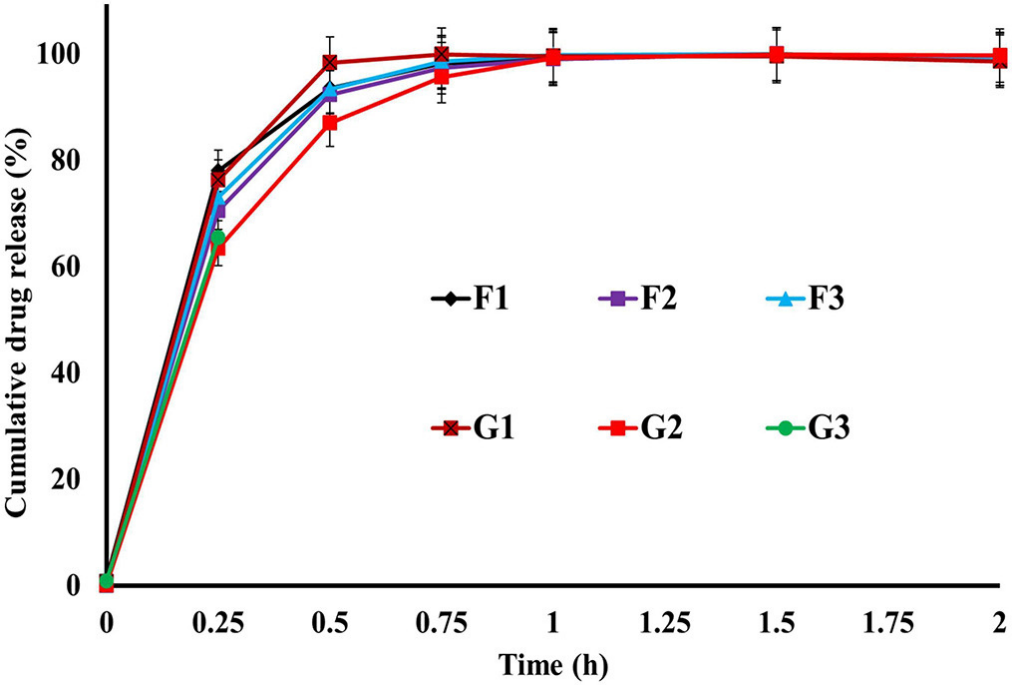
*In-vitro* drug release profile of R-BC-famotidine (F1–F3) and R-BC-tizanidine (G1–G3) matrices using USP type-I dissolution apparatus.

**Figure 7:**
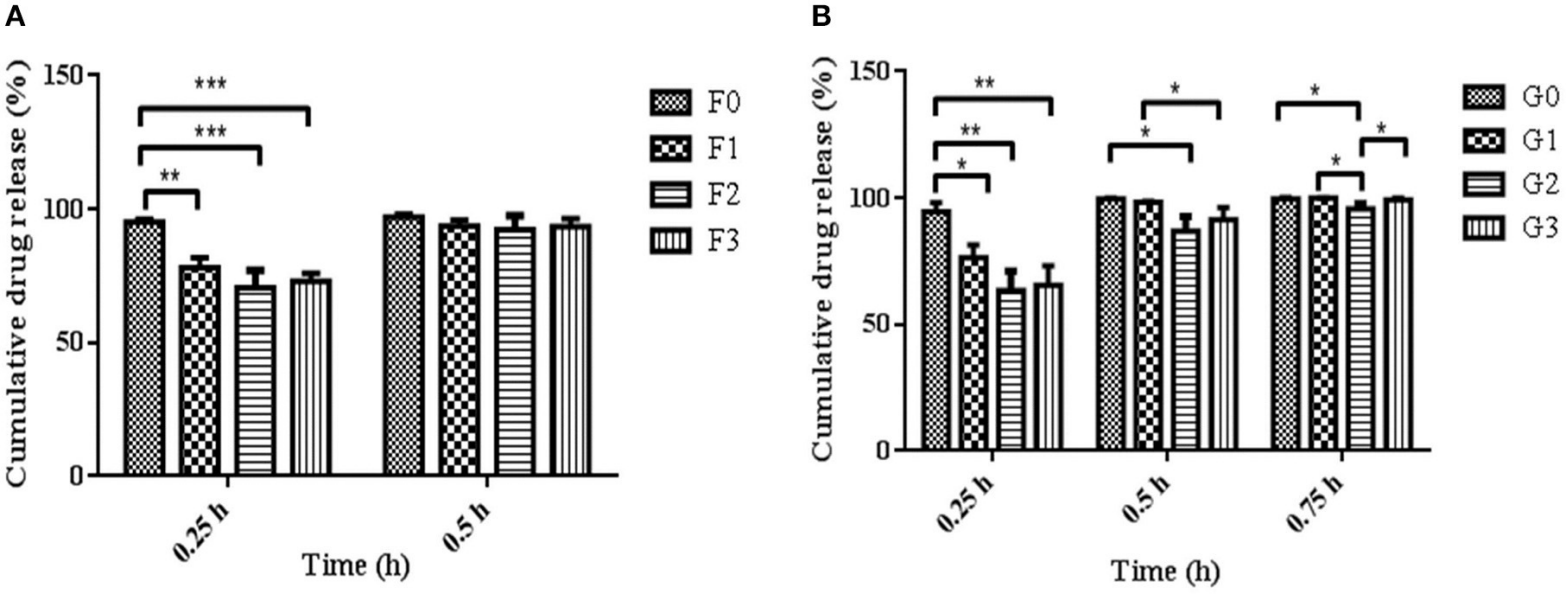
Comparison of R-BC matrices drug release data at various intervals of time i.e., **(A)** represent R-BC-famotidine matrices (F1–F3), at 0.25 and 0.5 h, whereas **(B)** denote R-BC-tizanidine matrices (G1–G3) at 0.25, 0.5, and 0.75 h, using one way ANOVA with *post-hoc* Tukey's test, keeping the level of significance with probabilities of *p < 0.05, **p < 0.01, and ***p < 0.001. Data is presented as mean ± SD (*n* =3).

The release of drugs from the R-BC-drug matrices was studied in simulated gastric fluid, i.e., 0.1 N HCl solution using USP type-I dissolution apparatus under predefined conditions. Figure 6 and Figure 7 show the *in-vitro* drug release profile from R-BC-famotidine matrices (F1–F3) and tizanidine loaded R-BC matrices (G1–G3). The matrices F1, F2, and F3 released 78.03 ± 3.77, 70.52 ± 6.52, and 73.04 ± 2.85% of the drug content, respectively, in the initial 15 min (*p* < 0.001) (Figure 7). The formulation F1 released higher concentration of drug than F2 and F3, whereas, no significant difference was observed between F2 and F3. Similarly, 93.57 ± 2.36, 92.28 ± 5.30, and 93.40 ± 3.00% of the drug was released has been shown by F1, F2, and F3 matrices after 30 min, which is not significant. However, at later time point i.e., 45 min, it was observed that regardless of the concentrations of loaded drug (Table 1), the matrices released most of the drug content (>95%), which was observed as 98.25 ± 0.78, 97.34 ± 2.99, and 98.58 ± 1.36% for F1, F2, and F3, respectively. In contrast to famotidine loaded matrices, the drug release from the R-BC-tizanidine matrices, i.e., G1, G2, and G3 was 76.260 ± 5.20, 63.40 ± 7.74, and 65.43 ± 7.73% at 15 min, respectively. These results reveals significant difference in drug release (*p* < 0.05) between formulations G1 with G2 and G3, whereas G2 and G3 have no significant difference. Moreover, 98.30 ± 0.4, 86.98 ± 5.59, and 91.33 ± 4.93% of the drug was released (*p* < 0.05), respectively, after 30 min from these matrices. This shows that G1 has released higher concentrations of the loaded drug as compared to G2 and G3. This behavior of comparatively slow drug release from G2 and G3 might be due to the higher concentrations of the drug (remained intact with R-BC) into matrices (Table 1). The R-BC-tizanidine matrices released most of the drug (>95%) after 45 min of studies and was found to be 99.91 ± 0.15, 95.65 ± 2.21, and 99.36 ± 0.72% for the formulations G1, G2, and G3 (*p* < 0.01), respectively. It was observed that t_90%_ (time in which 90% of the drug was released) is <1 h for both of the drug loaded matrices. The comparison of drug release from F1–F3 and G1–G3 show that famotidine loaded matrices have released higher concentrations of drug as compared to tizanidine in the initial 15 min. This might become possible due to larger exposed surface of R-BC for binding of the lower concentration of tizanidine as compared to famotidine loaded formulations (Kolakovic et al., 2013). However, after 30 min there have been no significant changes observed in drug release between F1–F3 and G1–G3. The possible reasons for the faster release of drugs from all formulations may be the aqueous solubility of the drugs (Badshah et al., 2017) and breakdown of the interconnected fibrous network hydrogen bonding of the BC as result of dissolution (Lee et al., 2014; Xu et al., 2015). R-BC has higher hydrophilic nature and absorbs aqueous solution faster. This feature might have facilitated the rapid penetration of dissolution medium into matrices, which resulted in faster dissolution and release of drugs (Gao et al., 2011; Badshah et al., 2017). In addition, the presence of loaded drug particles might have effected the film and pores formation (Figure 4) during the regeneration process, which have made the system strongly hydrophilic and thus facilitateed the rapid diffusion of dissolution medium into the matrices. These factors may resulted in the faster drug release from the matrices (Fink et al., 2001). Furthermore, the matrices washing for the removal of NMMO and freeze-drying may have facilitated the drug migration toward the surfaces, which has resulted in faster dissolution of the drugs and speedy release. This behavior might have facilitated the desired concentrations of the drugs to be released in the dissolution medium effectively (Isogai and Atalla, 1998; Ávila et al., 2015). It was also observed that early drug release behavior of R-BC have the potential for designing immediate release formulations (i.e., in stomach) based on single polymer. As mentioned above, the drug release is limited to few hours; however, the drug release could further be tailored by using other strategies with altered drugs aqueous solubility and their interactions with the BC nanofibers (Kolakovic et al., 2012). For statistical calculations of the effect of BC regeneration on the drug release from the mentioned formulations (F1–F3 and G1–G3), drug release results from as-synthesized BC matrices (F0 and G0) were used as reference (Figure 7).

It is evident from our current and previous studies that in comparison to the existing conventional dosage forms, BC forms a single excipient based intact oral dosage form due its higher tensile strength. Moreover, the as-synthesized BC membrane has limited thickness and more time is required to obtain desired thickness (Badshah et al., 2017; Ullah et al., 2017). In case of R-BC, the matrices with desired thickness can be easily produced by increasing the concentration of BC solution. Similarly, R-BC is more amorphous and easily biodegraded compared to as-synthesized BC and thus have high potential for drug delivery to the body parts, wherein degradation of BC is desired (Ullah et al., 2019).

### Drug Release Kinetics From the R-BC-Drug Matrices

The drug release kinetics studies revealed that the release of drugs from the R-BC-drug matrices was dependent on the drug concentration. The hydrophilic property of R-BC might facilitate the diffusion of the medium into the matrices. It was observed that drug release during dissolutions was best fitted into the first order kinetics model with *R*^2^ value more than 0.99. The release exponent “*n”* value of Korsmeyer-Pappas model was different for *in-vitro* dissolution. In case of the dissolution studies, the value of “*n”* was <0.5, which showed that release mechanism was following Fickian diffusion (Badshah et al., 2017, 2018) as presented in Table 2.

**Table 2:** Drug release kinetics of famotidine and tizanidine from the R-BC-drug matrices.

**Formulation**	**Zero order**		**First order**		**Higuchi**		**Korsmeyer**	
	***R*^**2**^**	**K_**0**_h^**−1**^**	***R*^**2**^**	**K_**1**_h^**−1**^**	***R*^**2**^**	**K_**H**_^**h−0.5**^**	***n***	***R*^**2**^**
F1	0.4896	72.276	0.9993	5.936	0.5722	91.901	0.101	0.9879
F2	0.3376	71.847	0.9997	4.935	0.6440	91.047	0.136	0.9771
F3	0.3753	72.206	0.9998	5.298	0.6208	91.750	0.122	0.9790
G1	0.5115	72.526	0.9980	6.025	0.5404	92.520	0.098	0.9757
G2	0.1349	71.326	0.9998	4.064	0.7337	89.766	0.182	0.9717
G3	0.2580	71.947	0.9978	4.510	0.6758	90.967	0.158	0.9626

## Conclusion

The current research work was carried out for the first time to evaluate the potential applications of regenerated BC for drug delivery. The R-BC-drug matrices were prepared using NMMO as solvent. Characterization data showed that R-BC-drug matrices were chemically and thermally stable. The drug loading and *in-vitro* drug release studies revealed that R-BC-drug matrices released more than 90% of the loaded amount of drugs in the initial 30 min during dissolution studies, which followed the criteria for immediate release drug delivery system. It can be concluded that R-BC, a novel and physically modified form of BC, has the ability for designing matrices for the delivery of drugs via oral route. However, further research work is required for the exploration of its potential applications in drug delivery via other routes of administration using other drugs.

## Data Availability Statement

The raw data supporting the conclusions of this article will be made available by the authors, without undue reservation.

## Author Contributions

TK, FW, and FH conceived the project, supervised the research, and writing of the manuscript. MB carried out the research work and wrote the manuscript basic draft in collaboration with HU and UF. MA has contributed in the characterization of samples and reviewed the manuscript critically. All authors contributed to the article and approved the submitted version.

## Conflict of Interest

The authors declare that the research was conducted in the absence of any commercial or financial relationships that could be construed as a potential conflict of interest.
