# Supplementary figures and images for: Development and Evaluation of Drug Loaded Regenerated Bacterial Cellulose-Based Matrices as a Potential Dosage Form
Source: Front Bioeng Biotechnol. 2020 Dec 3;8:579404. doi: 10.3389/fbioe.2020.579404 (PMC7744486; doi:10.3389/fbioe.2020.579404)

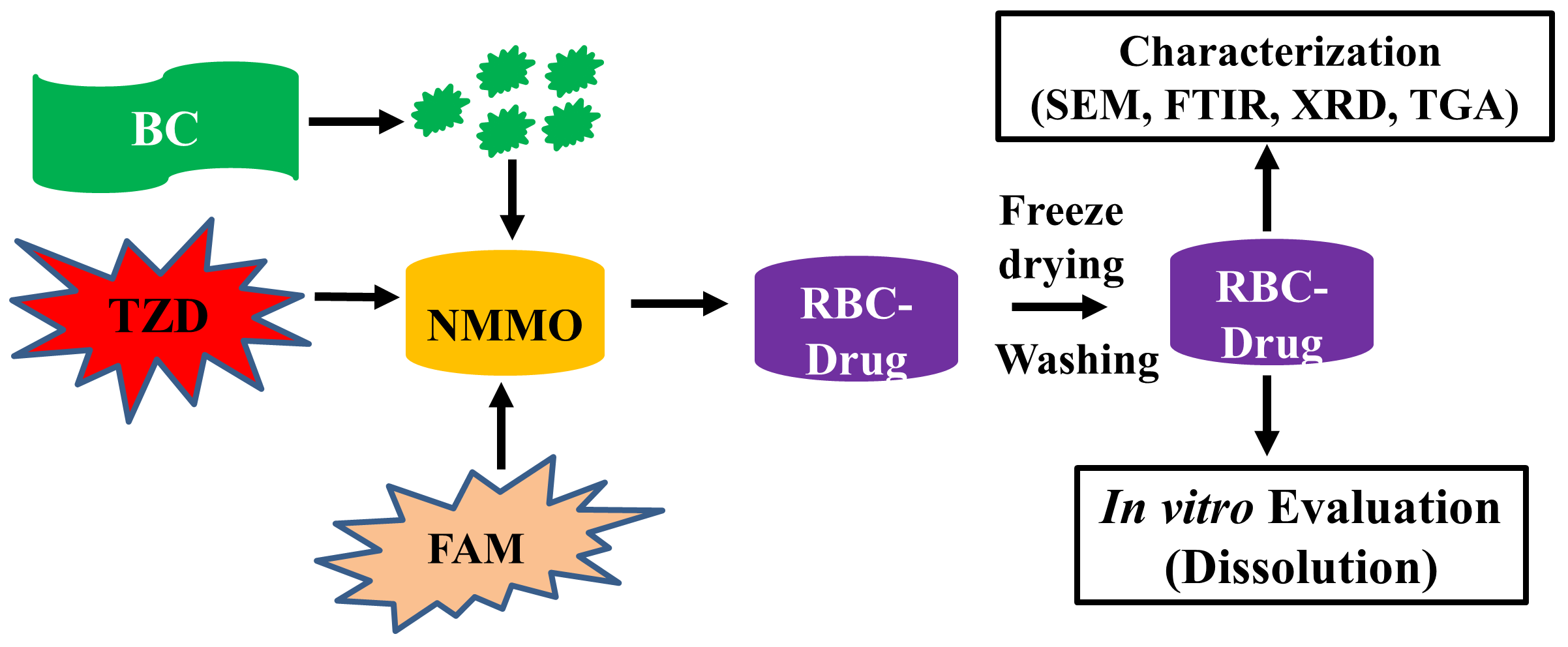

Supplement: Supplementary file 1 [file Image_1.TIF]
